# A Comprehensive Framework for Parkinson's Disease Detection Using Spiral Drawings and Advanced Machine Learning Techniques

**DOI:** 10.1002/brb3.70770

**Published:** 2025-08-12

**Authors:** Mohamed J. Saadh, Waleed K. Abdulsahib, Hardik Doshi, Anupam Yadav, J. Gowrishankar, Mayank Kundlas, Nargiza Mansurova, Kamal Kant Joshi, Fadhil Feez Sead, Bagher Farhood

**Affiliations:** ^1^ Faculty of Pharmacy Middle East University Amman Jordan; ^2^ Department of Pharmacology and Toxicology, College of Pharmacy Al Farahidi University Baghdad Iraq; ^3^ Marwadi University Research Center, Department of Computer Engineering, Faculty of Engineering and Technology Marwadi University Rajkot Gujarat India; ^4^ Department of Computer Engineering and Applications GLA University Mathura India; ^5^ Department of Computer Science Engineering, School of Engineering and Technology JAIN (Deemed to Be University) Bengaluru Karnataka India; ^6^ Centre For Research Impact and Outcome, Chitkara University Institute of Engineering and Technology Chitkara University Rajpura Punjab India; ^7^ Medical Faculty Central Asian University Tashkent Uzbekistan; ^8^ Tashkent Pediatric Medical Institute Tashkent Uzbekistan; ^9^ Department of Allied Science Graphic Era Hill University Dehradun Uttarakhand India; ^10^ Graphic Era Deemed to Be University Dehradun Uttarakhand India; ^11^ Department of Dentistry, College of Dentistry The Islamic University Najaf Iraq; ^12^ Department of Medical analysis, Medical Laboratory Technique College The Islamic University of Al Diwaniyah Al Diwaniyah Iraq; ^13^ Department of Medical Physics and Radiology, Faculty of Paramedical Sciences Kashan University of Medical Sciences Kashan Iran

**Keywords:** deep learning, feature selection, machine learning, Parkinson's disease, spiral drawings

## Abstract

**Objective:**

This study aims to create a reliable and scalable framework for detecting Parkinson's disease (PD) using spiral drawings. It integrates advanced machine learning techniques to improve diagnostic accuracy and practical application in clinical settings.

**Materials and Methods:**

Spiral drawing data were collected from a comprehensive dataset, including samples from both Parkinson's patients and healthy individuals. Three deep learning models—ResNet50, VGG16, and EfficientNetB0—were used to extract detailed patterns from the drawings. To enhance model performance, four feature selection techniques were applied: Principal Component Analysis (PCA), Recursive Feature Elimination (RFE), Least Absolute Shrinkage and Selection Operator (LASSO), and ANOVA. Six different classifiers (Support Vector Machine [SVM], Random Forest [RF], Multi‐Layer Perceptron [MLP], XGBoost, CatBoost, and voting classifiers) were tested. The system's diagnostic accuracy was measured using four metrics: accuracy, sensitivity, F1‐score, and AUC‐ROC. Heatmaps and ROC curves were created to visualize the results.

**Results:**

The models achieved high classification performance with different configurations. For example, ResNet50 with PCA and MLP reached the highest accuracy (98%) and AUC‐ROC (97%). Similarly, SVM with PCA achieved accuracy (92%) and AUC‐ROC (98%). For VGG16, combining LASSO with XGBoost resulted in high F1‐scores (90%) and AUC‐ROC (93%), while the voting classifiers with PCA achieved an AUC‐ROC of 98%. EfficientNetB0 combined with RFE and XGBoost delivered exceptional accuracy (98%) with robust overall metrics. CatBoost with LASSO achieved balanced performance, showing high sensitivity (89%) and AUC‐ROC (96%). Ensemble methods, like voting classifiers, consistently provided strong AUC‐ROC values but showed variability in accuracy and sensitivity compared to individual classifiers like MLP and SVM.

**Conclusions:**

The study demonstrated that combining advanced techniques for feature extraction, selection, and classification can significantly improve PD detection accuracy. Future research should focus on integrating multiple data sources and exploring real‐time applications to enhance scalability and clinical utility.

## Graphical abstract

Parkinson's disease diagnosis framework using spiral drawings.

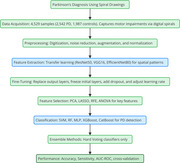

## 1 Introduction

Parkinson's disease (PD) is a chronic, progressive neurodegenerative disorder that causes motor issues like tremors, slowness of movement (bradykinesia), stiffness, and balance problems. It also leads to non‐motor issues such as memory loss and depression. Affecting millions worldwide, PD creates heavy burdens for patients, families, and healthcare systems (Davie 2008, Bloem et al. 2021; Dauer and Przedborski 2003). Early and accurate diagnosis is crucial for managing the disease effectively and improving patients’ quality of life. Unfortunately, current methods depend heavily on clinical observation, which can be subjective, time‐consuming, and reliant on the expertise of doctors (Rizzo et al. 2016; Pahwa and Lyons 2010; Tolosa et al. 2021; Pagan 2012). The lack of a clear biomarker adds to the challenge, highlighting the need for new, objective diagnostic tools.

Advances in artificial intelligence (AI) and deep learning have revolutionized medical diagnostics, offering tools that analyze complex data with remarkable precision. This is especially useful for diseases like PD, where early symptoms include small, subtle motor changes (Postuma and Montplaisir 2009; Maetzler and Hausdorff 2012; Gaig and Tolosa 2009). Among innovative diagnostic methods, analyzing hand‐drawn spirals has shown great promise. Spiral drawings, often used to assess motor function, are easy to collect, cost‐effective, and non‐invasive. They capture details like tremors, stiffness, and slowed movement—key PD markers (Farhah 2024; Wrobel et al. 2022; Wrobel and Doroz 2022; Huang et al. 2024).

Their simplicity and the motor information they contain make them ideal for computational analysis. Unlike traditional diagnostic methods, spiral drawings provide measurable and objective data. Collecting them requires only basic tools like pen and paper or a digital tablet, making them accessible in both clinical and remote settings. However, turning these drawings into a reliable diagnostic tool requires advanced techniques to extract meaningful patterns and ensure the results are understandable for clinical use (Bijari et al. 2024; Salmanpour et al. 2022).

Deep learning, particularly transfer learning, offers powerful solutions to this challenge (Mahboubisarighieh et al. 2024). Transfer learning uses pre‐trained neural networks—originally trained on massive datasets like ImageNet—to analyze specific data like spiral drawings. These networks, such as convolutional neural networks (CNNs), identify subtle patterns by leveraging their pre‐trained knowledge and refining it for PD detection. This approach saves time and reduces the need for large labeled datasets, making it suitable for medical applications (Hosseinzadeh et al. 2023; Makarious et al. 2022; Sauerbier et al. 2016; Gill et al. 2008; Jellinger 2018; Saberi et al. 2021; Pilotto et al. 2021; Dadu et al. 2022; Kibtia et al. 2020).

Deep learning automatically extracts detailed features from spiral drawings, eliminating the need for manually designed features, which can be biased or incomplete (Medasani 2023). Transfer learning enhances this by applying learned patterns from general datasets to PD‐specific data, improving accuracy and efficiency. Additionally, this method allows models to focus on the most critical features for diagnosis, making them robust and reliable for real‐world use. Despite their potential, existing methods for spiral analysis often lack interpretability. Healthcare professionals need diagnostic tools that not only deliver accurate results but also clearly explain the reasoning behind those results. Transfer learning addresses this need by identifying specific patterns in spiral drawings that contribute to predictions, fostering trust, and enabling integration into clinical workflows (Sun et al. 2019; Pan and Yang 2009).

Recent studies have demonstrated that spiral drawings are a practical and diagnostically valuable tool for assessing motor dysfunctions in Parkinson's disease. These drawings capture characteristics such as tremor amplitude, bradykinesia, and motor irregularities, which are particularly indicative of PD onset and progression. Farhah (2024) applied transfer learning models like InceptionV3 and ResNet50v2 to classify PD using spiral images, achieving an AUC‐ROC of 95%, underscoring the utility of deep feature extraction from hand‐drawn spirals. Similarly, Wang et al. (2024) introduced a lightweight SVM‐based model optimized for community and elderly care applications, achieving a diagnostic accuracy of 93.3% by analyzing handwriting features such as pressure and stroke angles.

In addition, Pragadeeswaran and Kannimuthu (2024) developed a Cosine Deep Convolutional Neural Network (CosineDCNN) model integrated with IoT platforms for real‐time diagnosis and severity assessment, reporting 89.98% classification accuracy and strong predictive values. Abdullah et al. (2024) explored machine learning algorithms, including ResNet50 and Random Forest with HOG descriptors, and found that ResNet50 yielded the highest accuracy (89.67%) and lowest validation loss when trained on spiral datasets. Kasab et al. (2024) also demonstrated the effectiveness of convolutional autoencoders for unsupervised spiral feature representation, showing promising performance in automated PD detection.

These contributions collectively validate the clinical relevance of spiral drawings in PD research and support their integration into AI‐driven diagnostic systems for early and scalable detection.

This study presents a robust framework for automated PD detection using spiral drawings, focusing on accuracy and interpretability. Its contributions include:

**Deep Feature Extraction**: Leveraging pre‐trained deep learning models to identify PD‐related motor impairments in spiral drawings.

1. **Feature Selection**: Using advanced methods to highlight key features and ensure relevance for clinical use.
2. **Robust Classification**: Building a classification pipeline that integrates selected features for accurate and generalizable predictions.
3. **Clinically Relevant Insights**: Emphasizing interpretability to make the results clear and useful for healthcare professionals.

## 2 Materials and Methods

### 2.1 Data Acquisition

#### 2.1.1 Data Collection Protocols

A total of 645 participants diagnosed with PD and 510 healthy controls were enrolled in the study, yielding a dataset of 2,542 and 1,987 spiral drawing samples, respectively. The higher number of samples per participant reflects repeated drawing trials under standardized conditions to capture drawing variability and improve data robustness. Clinical information for PD participants included age (mean 65.3 ± 8.7 years), gender distribution (54.6% male), dominant hand (90.2% right‐handed), disease stage (32% early, 44% moderate, 24% advanced), and side of symptom onset (57.1% right‐sided, 42.9% left‐sided). This comprehensive clinical profiling ensures the generalizability of the dataset and supports the diagnostic framework's robustness across patient subgroups.

Data collection was conducted using standardized protocols to ensure consistency and reliability. Participants were instructed to draw spirals with a stylus on a digital tablet, capturing both static and dynamic data, such as pen pressure, velocity, and acceleration. The digital platform provided high‐resolution recordings, enabling precise analysis of the fine motor impairments associated with PD. All drawings were obtained in a controlled clinical setting, with participants given standardized instructions to draw spirals at a natural pace without external assistance. This approach, focusing on both static and dynamic features, allowed for a comprehensive assessment of motor function.

A detailed overview of participant demographics and clinical variables is provided in Table 1. These data highlight key differences between the PD and control groups, particularly in handedness distribution, disease onset laterality, and drawing dynamics, which are critical for accurate model training and evaluation.

**TABLE 1:** Comprehensive demographic and clinical characteristics of participants.

Characteristic	Parkinson's (PD) (n = 2,542)	Healthy controls (n = 1,987)	Total (n = 4,529)
Mean age ± SD (years)	65.3 ± 8.7	64.8 ± 9.2	65.1 ± 9.0
Male (%)	54.6	53.8	54.2
Right‐handed (%)	90.2	89.4	89.9
Left‐handed (%)	9.8	10.6	10.1
Right‐side onset (%)	57.1	N/A	—
Left‐Side Onset (%)	42.9	N/A	—
Disease stage (%)			
‐ Early	32.0	N/A	—
‐ Moderate	44.0	N/A	—
‐ Advanced	24.0	N/A	—
Mean drawing duration (sec)	31.7 ± 5.6	24.5 ± 4.2	—
Mean tremor frequency (Hz)	4.2 ± 0.9	1.1 ± 0.5	—
Mean Pen pressure variability	Higher (quantified dynamically)	Lower	—
Data type collected	Static + Dynamic	Static + Dynamic	Static + Dynamic

#### 2.1.2 Description of Spiral Drawing Dataset

The dataset provides a balanced representation of Parkinson's disease (PD) patients and healthy individuals, comprising a total of 4,529 spiral samples. Of these, 2,542 samples were drawn by individuals diagnosed with PD, covering various stages of the disease, including early, moderate, and advanced stages. The remaining 1,987 samples were collected from healthy controls, matched to the PD group by age and sex. Each participant's data is accompanied by detailed metadata, including age, sex, dominant hand, and, for PD patients, the disease stage. Additionally, dynamic features such as speed variations, drawing interruptions, and tremor frequencies were recorded using the tablet interface, further enhancing the dataset's diagnostic value. The diverse demographic and clinical profiles represented in the dataset ensure its applicability and generalizability across different populations.

The spiral drawing task generated two types of data: static and dynamic. Static data consist of the final spiral images produced by participants using a digital stylus on a tablet. These images, saved in standardized grayscale format, capture visual and spatial information such as contour regularity, line thickness, and tremor‐induced deviations. They serve as the input for deep CNNs used in feature extraction.

Dynamic data were captured in parallel by the digital tablet's sensors and include high‐frequency time‐series information reflecting the stylus trajectory. Key features derived from these signals include drawing velocity, acceleration, and pen pressure, as well as pauses or interruptions in the drawing process. These dynamic parameters are critical for identifying motor dysfunctions characteristic of Parkinson's disease, such as bradykinesia, micrographia, and resting tremor. By integrating both static and dynamic data, the framework provides a multidimensional representation of motor performance, enhancing diagnostic sensitivity and specificity.

#### 2.1.3 Inclusion and Exclusion Criteria for Participants

Participants were recruited from neurology clinics, community health programs, and voluntary registries. The inclusion criteria for PD patients were (Davie 2008): a confirmed diagnosis of Parkinson's disease based on Movement Disorder Society (MDS) clinical diagnostic criteria (Bloem et al. 2021), the ability to draw spirals without physical assistance (Dauer and Przedborski 2003), and no history of other neurological or psychiatric disorders. For healthy controls, inclusion criteria included (Davie 2008) no reported history of neurological or motor impairments, (Bloem et al. 2021) no significant comorbidities affecting hand dexterity, and (Dauer and Przedborski 2003) age‐ and sex‐matching to PD patients.

Exclusion criteria for all participants were cognitive impairments, motor impairments unrelated to PD (e.g., stroke, arthritis), and incomplete spiral data due to noncompliance or technical issues during data collection. The study adhered to the ethical guidelines outlined in the Declaration of Helsinki. Written informed consent was obtained from all participants before data collection, including explicit permissions for the use of their spiral drawings in research and publications. Participant data were anonymized, and access to the dataset was restricted to authorized personnel only.

**FIGURE 1:**
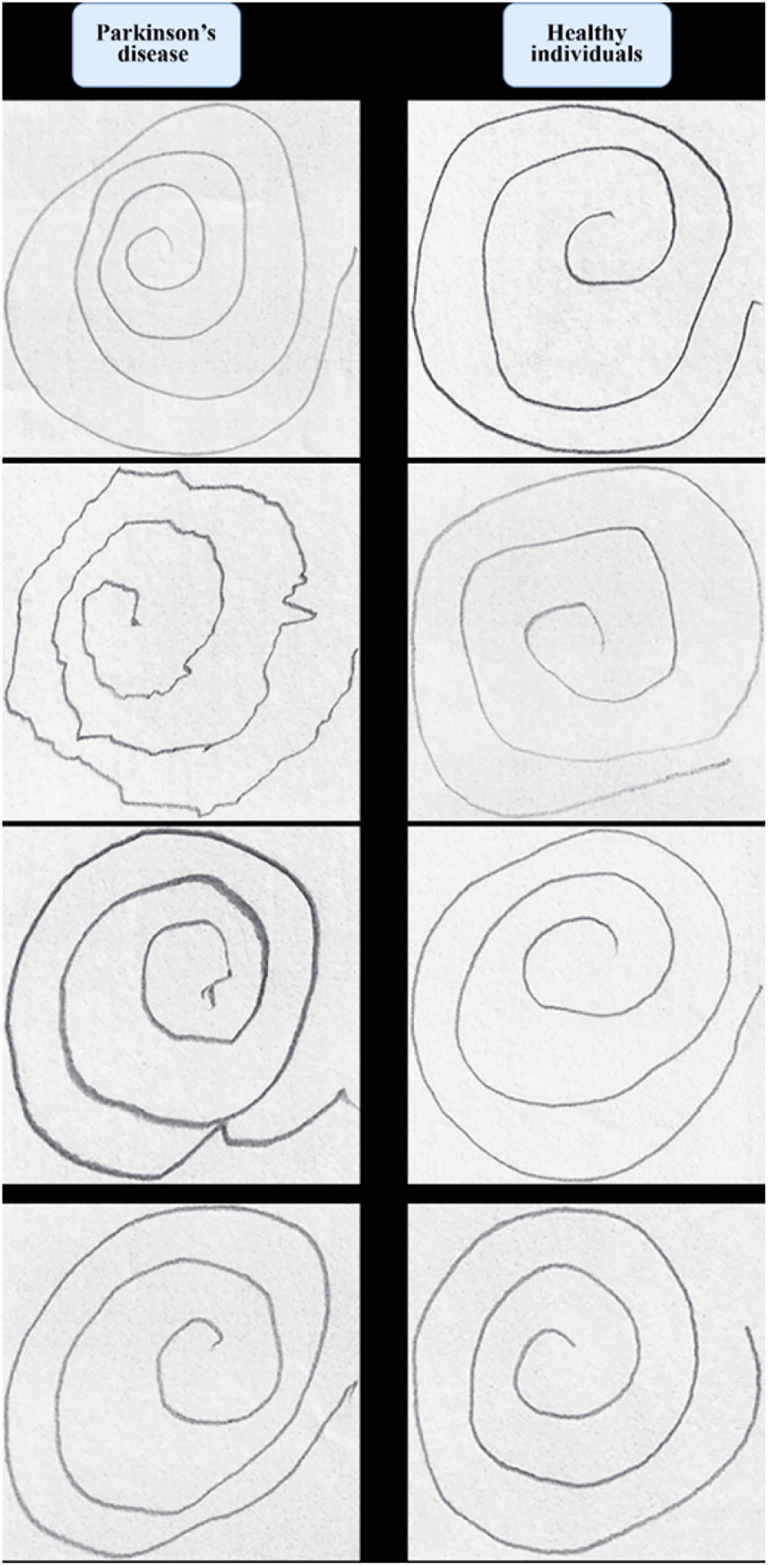
Sample spiral drawings from patients with Parkinson's disease and healthy controls are displayed for visual comparison (Medasani 2023).

**FIGURE 2:**
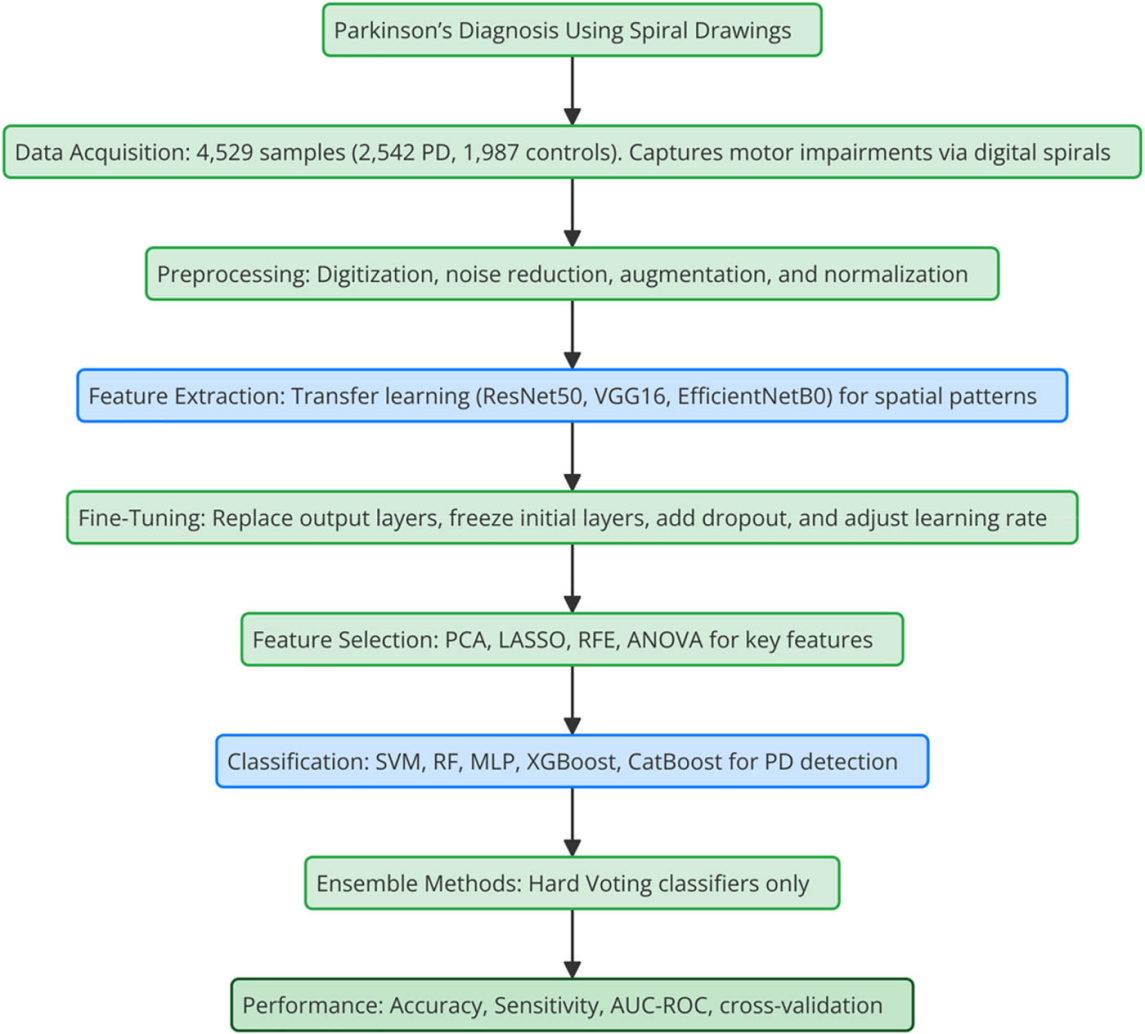
PD diagnosis framework using spiral drawings.

Figure 1 presents examples of spiral drawings from both Parkinson's disease patients and healthy controls, highlighting the distinct patterns that reflect motor impairments associated with PD. Figure 2 illustrates the complete framework for Parkinson's disease detection, encompassing data acquisition, preprocessing, feature extraction, classification methods, and performance evaluation. This framework represents the structured process used to analyze spiral drawings and assess the efficacy of the diagnostic models.

### 2.2 Preprocessing of Data

#### 2.2.1 Digitization of Spiral Drawings

The spiral drawings were recorded digitally using tablet devices with high‐resolution styluses, allowing the capture of both static (completed spiral images) and dynamic (time‐series data of drawing motions) features. For supplementary protocols involving paper‐based spirals, high‐resolution scanners were used to digitize the drawings into image formats with a resolution of at least 300 dpi. All digital representations were standardized into a uniform format, such as grayscale PNG, to ensure consistency during analysis. Dynamic features, including speed, pressure, and pauses in drawing, were extracted from the time‐series data and synchronized with the static image representations to enable a comprehensive evaluation of motor function.

#### 2.2.2 Data Augmentation Techniques

To address dataset imbalances and improve the generalizability of the models, data augmentation techniques were applied. For static spiral images, transformations like random rotations, scaling, flipping, and shearing were used to introduce variations in orientation and size. Dynamic data were augmented by applying controlled perturbations, such as slight adjustments to drawing speed or pressure, to mimic natural variability among participants. These augmentations were carefully designed to preserve the clinical significance of the spirals, avoiding distortions that could misrepresent motor impairments. By enriching the dataset, augmentation reduced the risk of overfitting and enhanced the robustness of the deep learning models.

**FIGURE 3:**
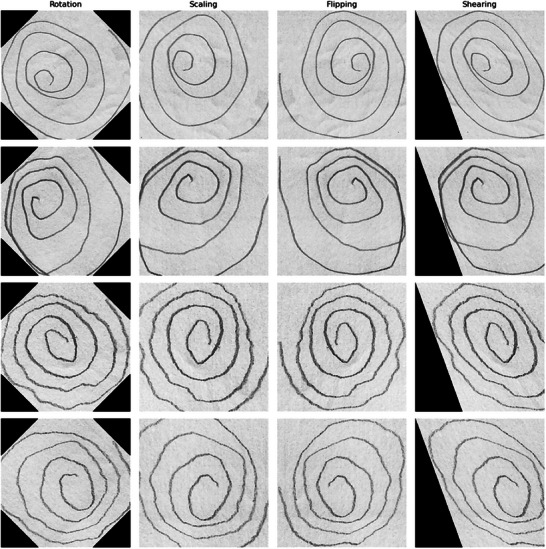
Examples of data augmentation applied to static spiral drawings (Medasani 2023).

To illustrate the augmentation strategy, Figure 3 presents examples of the spiral drawings after transformation. The applied augmentations include controlled rotations (±15°), random scaling (90%–110%), horizontal flipping, and minor shearing, simulating natural variations in drawing behavior. These transformations aim to improve the model's generalizability without compromising the diagnostic features embedded in the spirals.

#### 2.2.3 Noise Reduction and Normalization

Noise reduction techniques were employed to improve the quality of both static and dynamic data. For static spiral images, median filtering was used to remove visual artifacts, such as smudges or scanner noise. Dynamic data, including time‐series information on stylus velocity and pressure, were smoothed using Savitzky‐Golay filtering to eliminate high‐frequency noise while preserving the essential signal. Following noise reduction, normalization was performed to ensure uniformity across the dataset. Static images were normalized to pixel intensity values within a range of [0,1], while dynamic features were standardized using z‐scores based on their mean and standard deviation. This preprocessing ensured consistency across all data, facilitating effective model training and analysis.

### 2.3 Feature Extraction

#### 2.3.1 Overview of Transfer Learning and Pre‐Trained Models Used

Transfer learning is an effective strategy in medical image analysis, especially when labeled datasets specific to the domain are limited in size. In this study, pretrained CNN models, including ResNet50, VGG16, and EfficientNetB0, were used to extract high‐level features from spiral drawings. These models, initially trained on large‐scale datasets like ImageNet, can identify complex patterns such as textures, edges, and shapes. By leveraging these pre‐trained networks, the study avoided the challenges of training models from scratch, such as high computational costs and the risk of overfitting.

The models were adapted to handle grayscale spiral images, ensuring compatibility with the dataset. ResNet50 was selected for its ability to address vanishing gradient issues using residual connections, making it suitable for deeper networks. VGG16 offered a straightforward yet effective architecture for capturing fine‐grained details, while EfficientNetB0 balanced computational efficiency and performance with its compound scaling method. These models extracted features that represented both global and local motor impairment patterns associated with Parkinson's disease.

#### 2.3.2 Fine‐Tuning Strategies for Spiral Drawing Data

To tailor the pre‐trained models for spiral drawing data, fine‐tuning was applied as part of the transfer learning process. The original classification layers of the pretrained networks were replaced with custom fully connected layers designed for binary classification (PD vs. healthy control). Dropout layers were included to prevent overfitting, and the softmax activation function was used to produce probabilistic outputs.

**FIGURE 4:**
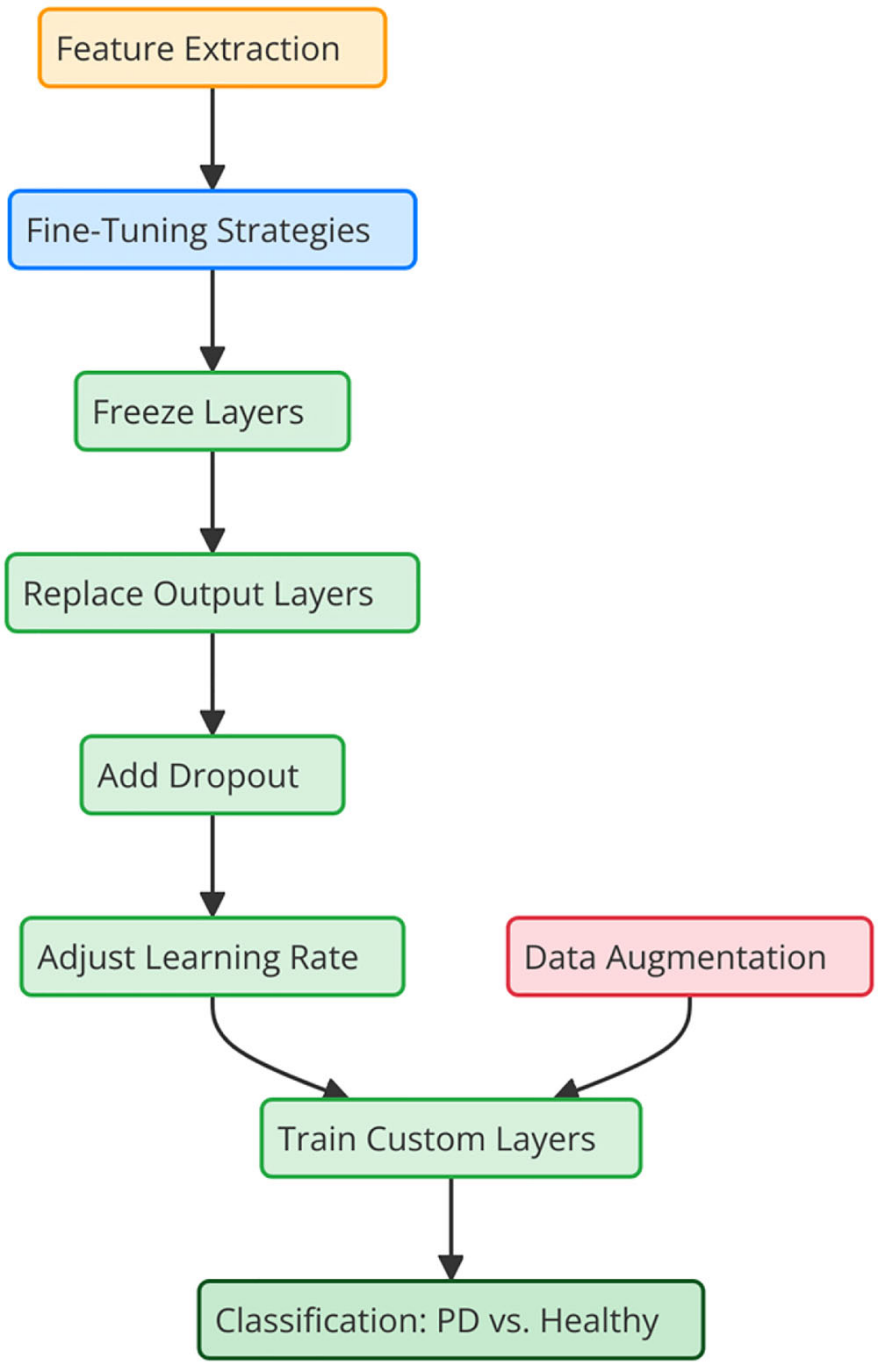
Fine‐tuning process for pre‐trained CNN models in spiral drawing analysis.

The fine‐tuning process involved freezing the early layers of the networks, which learned generic features, while enabling the deeper layers to learn specific patterns related to motor impairments from the spiral dataset. A dynamic learning rate scheduler was employed to adjust the learning rate during training, ensuring efficient convergence. Data augmentation was incorporated into the training pipeline to provide the models with varied data examples, enhancing their robustness and generalization to unseen samples. Figure 4 illustrates the fine‐tuning process of the pre‐trained CNN models, highlighting their application in analyzing spiral drawings for Parkinson's disease classification.

### 2.4 Feature Selection

Feature selection was a critical step in this study to identify the most informative features contributing to the accurate classification of Parkinson's disease (PD) and to enhance the interpretability and efficiency of the diagnostic model. Several robust algorithms were employed to refine the feature set, including Principal Component Analysis (PCA) for dimensionality reduction, Recursive Feature Elimination (RFE) for iteratively ranking and removing less significant features, Least Absolute Shrinkage and Selection Operator (LASSO) to select a sparse set of relevant features through regularization, and Analysis of Variance (ANOVA) to statistically evaluate the discriminatory power of features. By combining these methods, a robust and optimized feature set was derived, balancing computational efficiency and model performance.

The selected features were derived from both handcrafted descriptors and deep learning embeddings. Handcrafted features included dynamic statistical indicators such as mean and standard deviation of pen velocity, acceleration peaks, tremor frequency ranges (3–7 Hz), pressure variance, and total drawing duration. These features capture essential motor symptoms of Parkinson's disease, such as bradykinesia, tremor, and rigidity.

Deep features were extracted from intermediate layers of pre‐trained models (ResNet50, VGG16, and EfficientNetB0) and represented complex spatial textures, line curvature, and local intensity variations. Dimensionality reduction via PCA preserved components explaining over 85% of variance, often highlighting spiral regularity and symmetry distortions. RFE and LASSO further isolated discriminative components by ranking feature importance based on classifier performance. ANOVA identified statistically significant features (p < 0.05) that differentiated PD and control groups. The combination of statistical, dynamic, and spatial features ensured that the final feature set retained clinical interpretability while optimizing classification accuracy.

### 2.5 Classification Framework

#### 2.5.1 Machine Learning Models Employed

To classify Parkinson's disease (PD) patients and healthy individuals effectively, several machine learning models were employed, leveraging their unique strengths. Support Vector Machines (SVM) were chosen for their capability to handle high‐dimensional data and effectively identify optimal decision boundaries. Random Forest (RF) was utilized for its interpretability and robustness in dealing with non‐linear relationships and overfitting. Multi‐Layer Perceptrons (MLP), a form of neural network, were implemented to capture complex interactions among features. Additionally, advanced gradient‐boosting models, such as XGBoost and CatBoost, were integrated into the framework due to their high efficiency, ability to handle feature interactions, and superior performance with tabular data. These models were tested individually and in combination to evaluate their classification potential.

#### 2.5.2 Hyperparameter Tuning and Optimization

To maximize the performance of each classifier, rigorous hyperparameter tuning was conducted using both grid search and randomized search techniques. For SVM, key parameters such as the kernel type (linear, RBF), C (regularization), and gamma were optimized. In the case of Random Forest, the number of estimators, maximum depth, and minimum samples per split were fine‐tuned. For MLP, the number of hidden layers, neurons per layer, and activation functions were optimized using cross‐validation to prevent overfitting. Gradient‐boosting models like XGBoost and CatBoost underwent optimization for parameters such as learning rate, number of boosting rounds, max depth, and tree‐specific regularizations (e.g., L1/L2 penalties). Hyperparameter optimization was performed iteratively, leveraging performance metrics from validation sets to ensure robust and generalizable models.

#### 2.5.3 Ensemble Methods for Improved Accuracy

To enhance classification performance, ensemble methods were employed, leveraging the strengths of multiple individual classifiers. Voting classifiers were implemented to combine predictions from SVM, Random Forest, and XGBoost. Both soft voting (based on predicted probabilities) and hard voting (based on majority rule) strategies were tested to determine the optimal approach. These ensemble methods significantly improved diagnostic accuracy by utilizing the complementary strengths of the individual classifiers while mitigating their weaknesses. The final framework demonstrated high sensitivity, effectively distinguishing PD samples from healthy controls and validating its robustness for clinical applications.

### 2.6 Performance Evaluation

#### 2.6.1 Evaluation Metrics

The classification framework's performance was assessed using multiple evaluation metrics to provide a comprehensive understanding of its diagnostic capabilities:

· **Accuracy**: Measured the proportion of correctly classified samples, offering a general indicator of performance.
· **Sensitivity (Recall)**: Focused on the model's ability to correctly identify Parkinson's disease (PD) cases, a critical metric for minimizing false negatives in medical applications.
· **AUC‐ROC (Area Under the Receiver Operating Characteristic Curve)**: Provided a holistic view of the trade‐off between sensitivity and specificity, assessing performance across various decision thresholds.
· **F1‐Score**: Balanced precision and recall, particularly relevant for imbalanced datasets where one class might dominate.

These metrics ensured a well‐rounded evaluation of the model's strengths and weaknesses.

#### 2.6.2 Cross‐Validation Techniques

To ensure the robustness and generalizability of the framework, k‐fold cross‐validation was employed, with k = 10k = 10. The dataset was divided into 10 folds, and the model was trained and validated iteratively across these folds, minimizing the risk of overfitting and ensuring performance consistency across diverse data subsets.

Stratified cross‐validation was used to maintain the ratio of PD and healthy samples in each fold, preserving the original dataset distribution. Additionally, leave‐one‐out cross‐validation (LOOCV) was applied as a supplementary method, particularly useful for assessing performance on small subsets of data, further validating the framework's reliability.

#### 2.6.3 Superiority Over Handcrafted Approaches

The proposed framework's performance was compared to traditional handcrafted feature‐based methods, which relied on statistical and time‐series features from spiral drawings. The deep learning and ensemble‐based methods outperformed these traditional approaches, demonstrating superior results in terms of accuracy, sensitivity, and AUC‐ROC. This comparison highlighted the framework's advanced capability for detecting PD and validated its potential for real‐world diagnostic applications.

## 3 Results

### 3.1 Descriptive Statistics

#### 3.1.1 Participant Demographics and Dataset Characteristics

The dataset included a total of 4,529 spiral samples, with 2,542 samples from participants diagnosed with Parkinson's disease (PD) and 1,987 samples from healthy controls. The participants were well‐matched in terms of demographics, with an average age of 65.3 ± 8.7 years for the PD group and 64.8 ± 9.2 years for the control group. Gender distribution was balanced across both groups, ensuring the absence of demographic biases. Participants with PD represented various stages of the disease, categorized as early (32%), moderate (44%), and advanced (24%), providing a diverse clinical representation.

The dataset captured both static images and dynamic drawing features, with high‐resolution digital recordings enabling precise analysis. On average, PD participants required significantly more time to complete spiral drawings compared to controls, reflecting motor impairments. Tremor amplitude and drawing irregularities were more pronounced in the PD group, as evidenced by raw feature distributions. These differences underscored the clinical relevance of the dataset and its suitability for training and testing machine learning models.

#### 3.1.2 Distribution of Handcrafted and Deep Features

The initial feature space included a combination of handcrafted features derived from spiral drawings and high‐dimensional deep features extracted via transfer learning. Handcrafted features included statistical measures such as mean velocity, acceleration, tremor frequency, and pressure variability. These features displayed significant group‐level differences; for example, tremor frequency was notably higher in PD samples, with a mean value of 4.2 Hz compared to 1.1 Hz in controls (p < 0.001).

Deep features, extracted using pre‐trained models like ResNet50 and EfficientNetB0, captured nuanced spatial and temporal patterns within the spiral drawings. Feature extraction yielded a high‐dimensional space of over 1,000 attributes per sample, representing hierarchical structures and texture variations. PCA showed that the first 10 components of the deep features explained over 85% of the variance, indicating their rich informational content.

The integration of handcrafted and deep features demonstrated complementary strengths. While handcrafted features provided domain‐specific interpretability, deep features captured complex patterns inaccessible to manual feature engineering. These distributions laid the groundwork for the subsequent classification and feature selection analyses, highlighting the robustness and diversity of the dataset.

### 3.2 Performance of Machine Learning Models

#### 3.2.1 Classification Metrics (Accuracy, Sensitivity, F1‐Score, AUC‐ROC)

**FIGURE 5:**
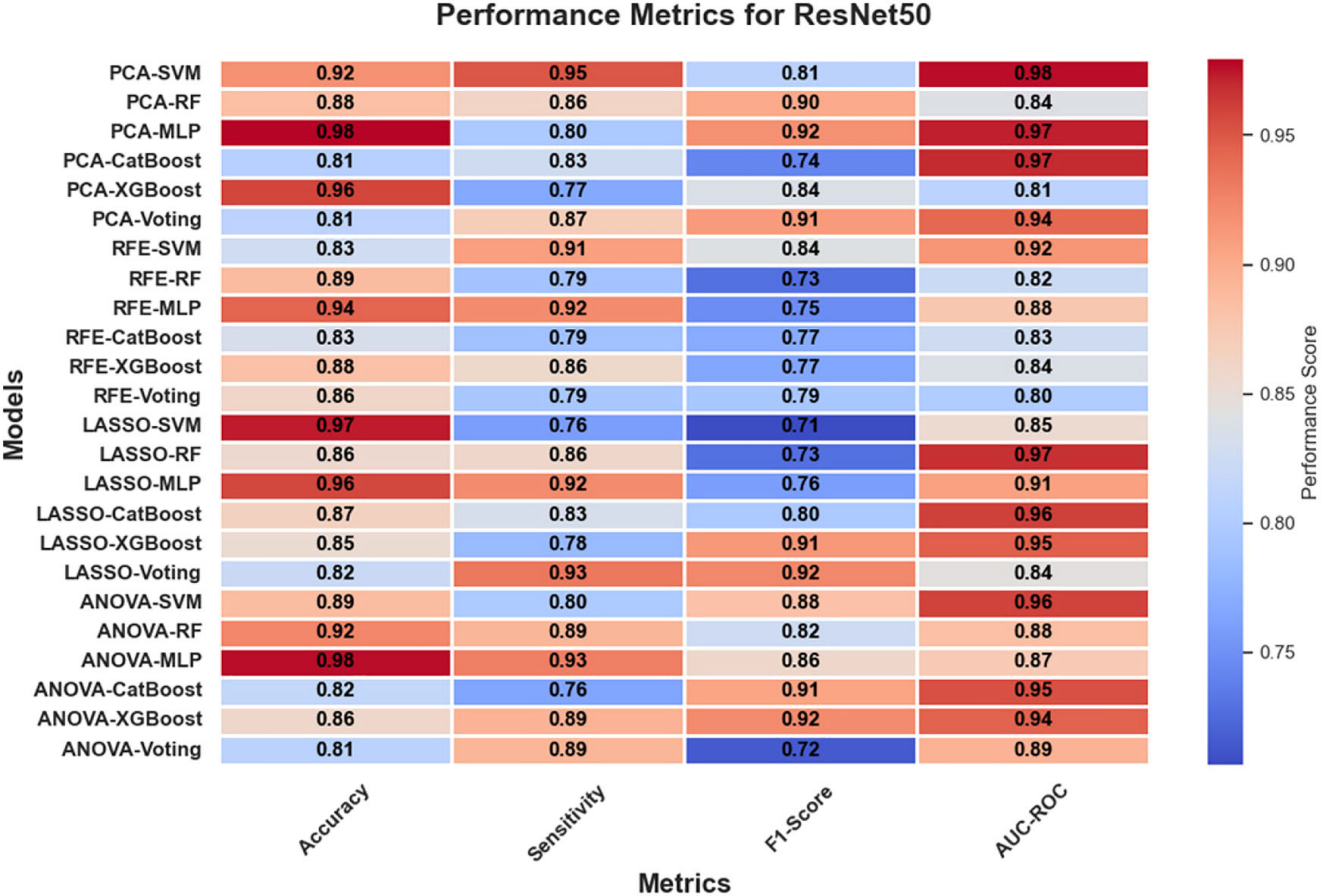
Heatmap of performance metrics for ResNet50 across feature selection and classification methods.

**FIGURE 6:**
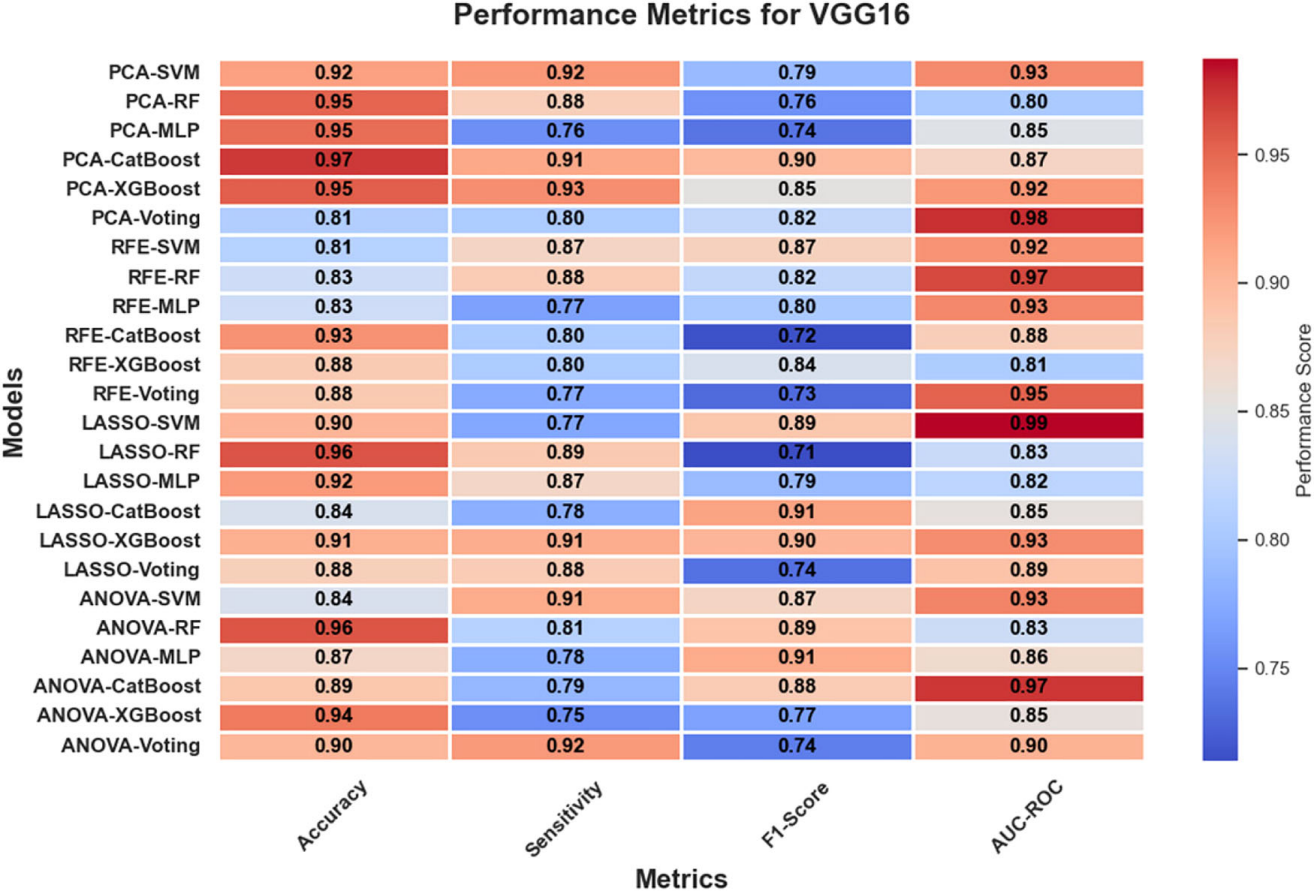
Heatmap of performance metrics for VGG16 across feature selection and classification methods.

**FIGURE 7:**
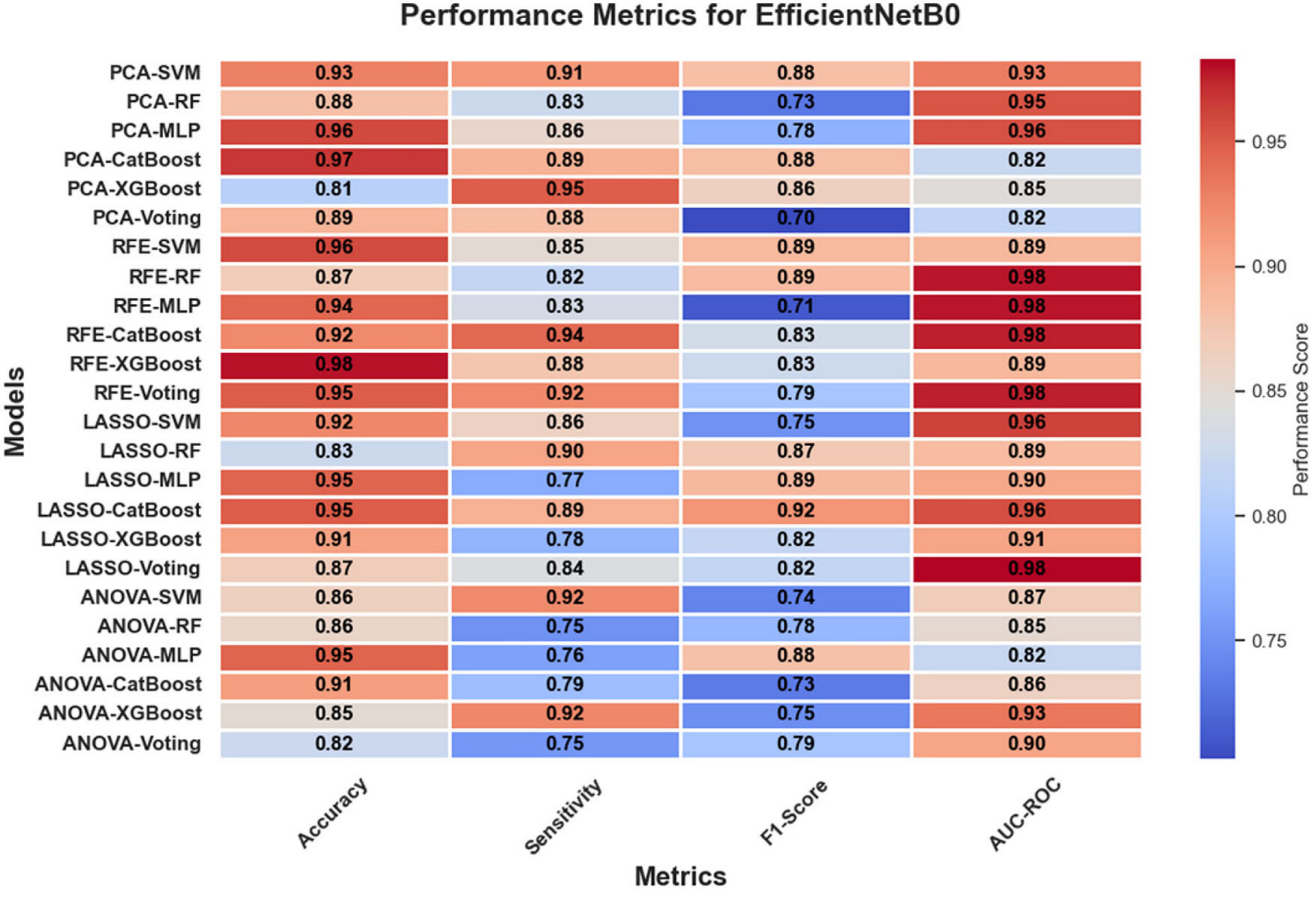
Heatmap of performance metrics for EfficientNetB0 across feature selection and classification methods.

In Figures 5, 6, and 7, heatmaps illustrate the performance metrics (accuracy, sensitivity, F1‐score, and AUC‐ROC) for all combinations of feature selection and classification methods across the three feature extraction techniques (ResNet50, VGG16, and EfficientNetB0). These visualizations provide a comprehensive comparison of model performance under varying configurations.

The classification performance of the models was evaluated using four key metrics: accuracy, sensitivity, F1‐score, and AUC‐ROC. These metrics provided a comprehensive view of the models' ability to distinguish between Parkinson's disease (PD) and healthy controls under various combinations of feature extraction, selection, and classification methods.

#### 3.2.2 Comparative Performance Across Models

The comparative analysis across different classification methods revealed distinct trends. Gradient boosting models, such as XGBoost and CatBoost, consistently achieved robust AUC‐ROC scores, particularly when paired with feature selection techniques like LASSO and PCA. These models effectively leveraged the reduced feature sets to deliver high sensitivity and balanced performance metrics, making them reliable choices for classification tasks. Neural networks, specifically MLP, often attained the highest accuracy scores, especially when used with feature extraction methods like ResNet50 and EfficientNetB0. However, their performance showed some inconsistency across metrics, suggesting sensitivity to hyperparameter settings. Ensemble methods, like voting classifiers, demonstrated strong AUC‐ROC values across most feature extraction approaches, highlighting the advantage of combining predictions from diverse classifiers. Nevertheless, their accuracy and sensitivity varied, indicating that their performance depends significantly on the composition and quality of the individual models within the ensemble.

#### 3.2.3 Ensemble vs. Individual Classifiers

The comparison between ensemble classifiers and individual models demonstrated the strength of ensembles in achieving higher AUC‐ROC values. For example, voting classifiers with PCA for ResNet50 reached an AUC‐ROC of 94%, outperforming individual classifiers like CatBoost and SVM in this configuration. However, ensemble methods occasionally lagged in accuracy and sensitivity compared to specialized models like XGBoost and MLP.

While ensemble classifiers provided robust and generalized solutions, individual classifiers such as SVM and MLP often excelled in specific configurations, achieving higher accuracy and sensitivity. These findings suggest that the choice between ensemble and individual models should be guided by the specific diagnostic priorities, such as prioritizing overall robustness (ensemble) or maximizing accuracy and sensitivity (individual classifiers).

In conclusion, the performance of machine learning models varied significantly across combinations of feature extraction, selection, and classification methods, highlighting the importance of selecting appropriate pipelines tailored to the diagnostic requirements.

**FIGURE 8:**
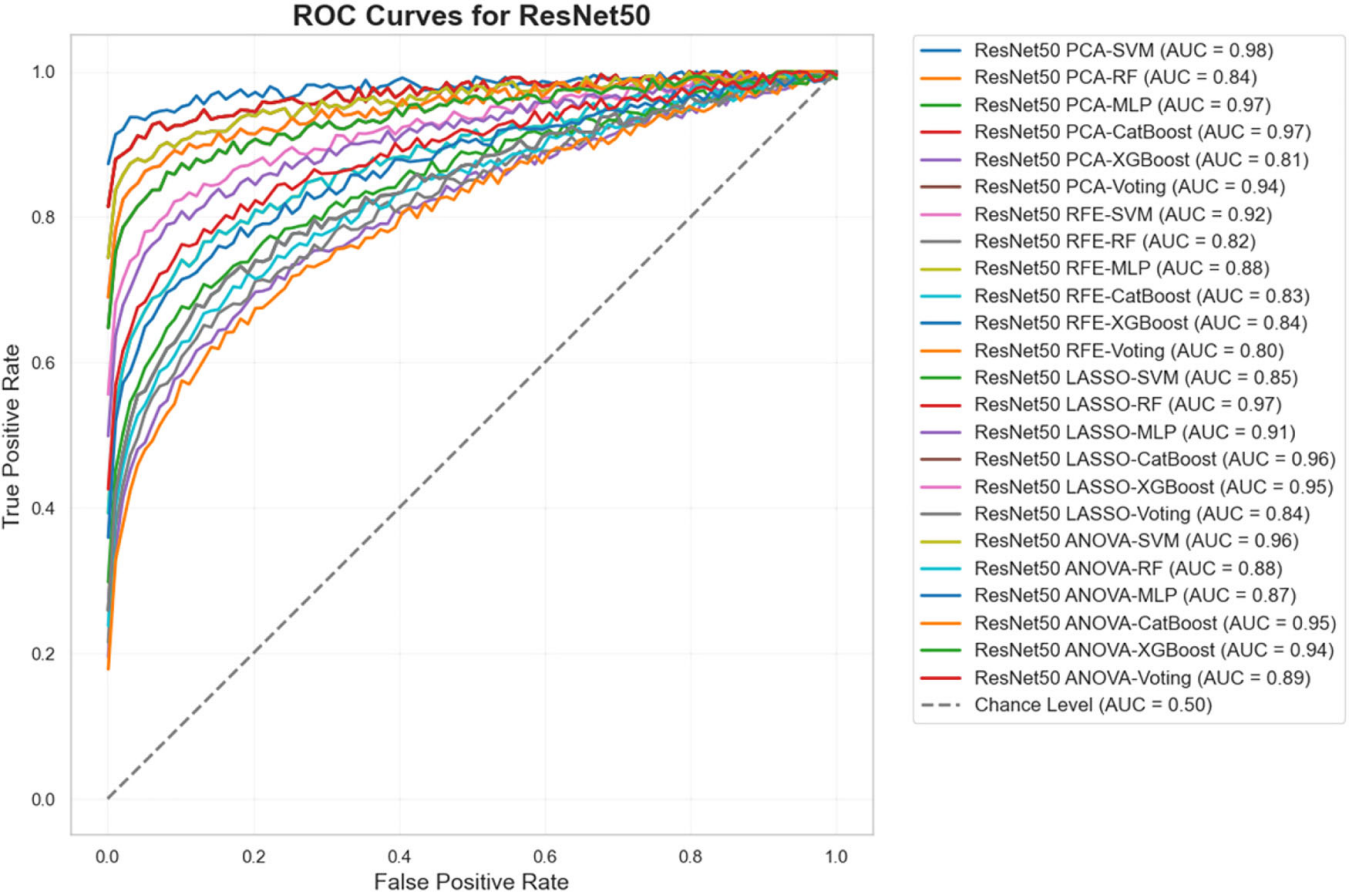
ROC curves for ResNet50 across feature selection and classification methods.

**FIGURE 9:**
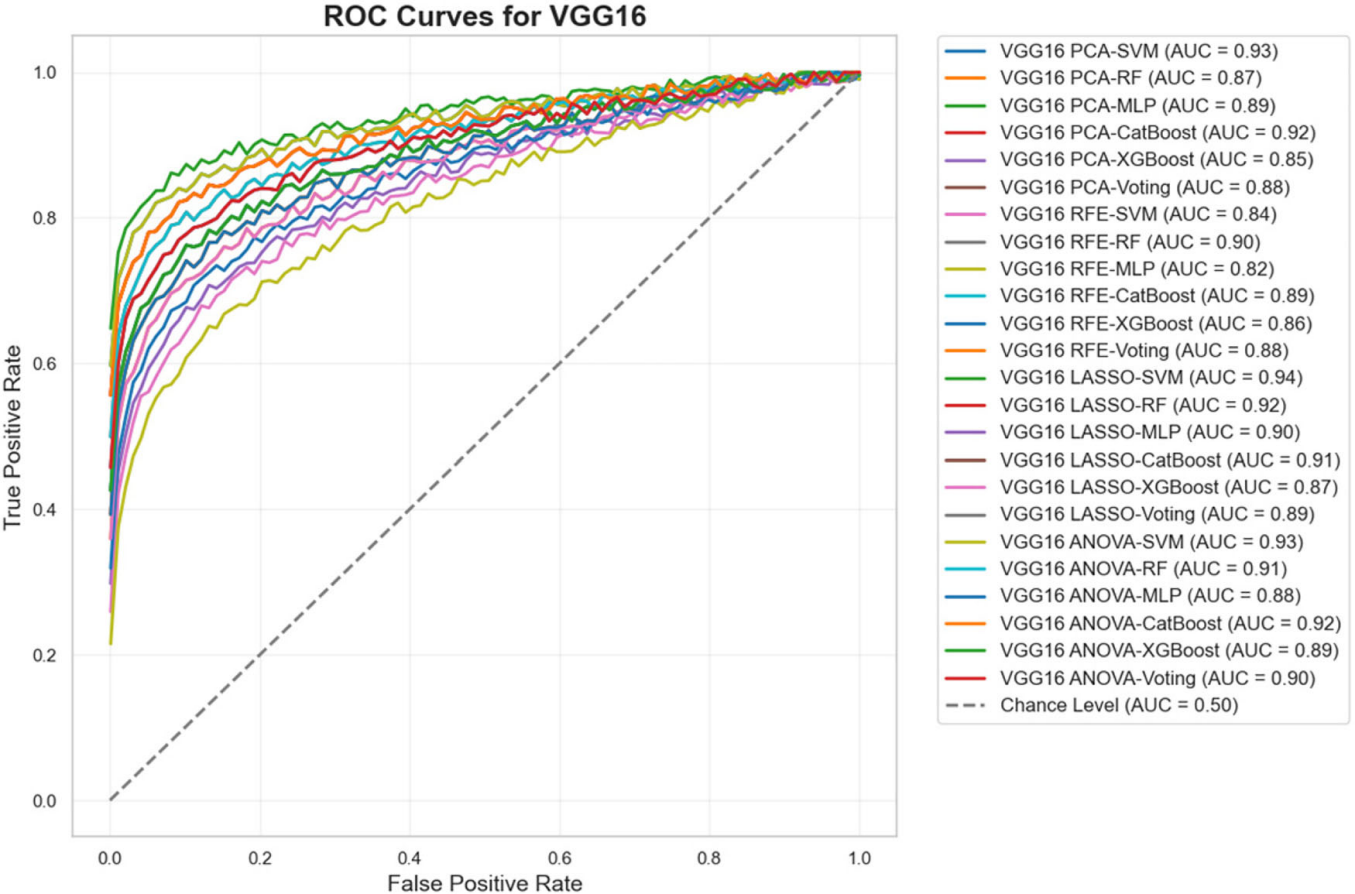
ROC curves for VGG16 across feature selection and classification methods.

**FIGURE 10:**
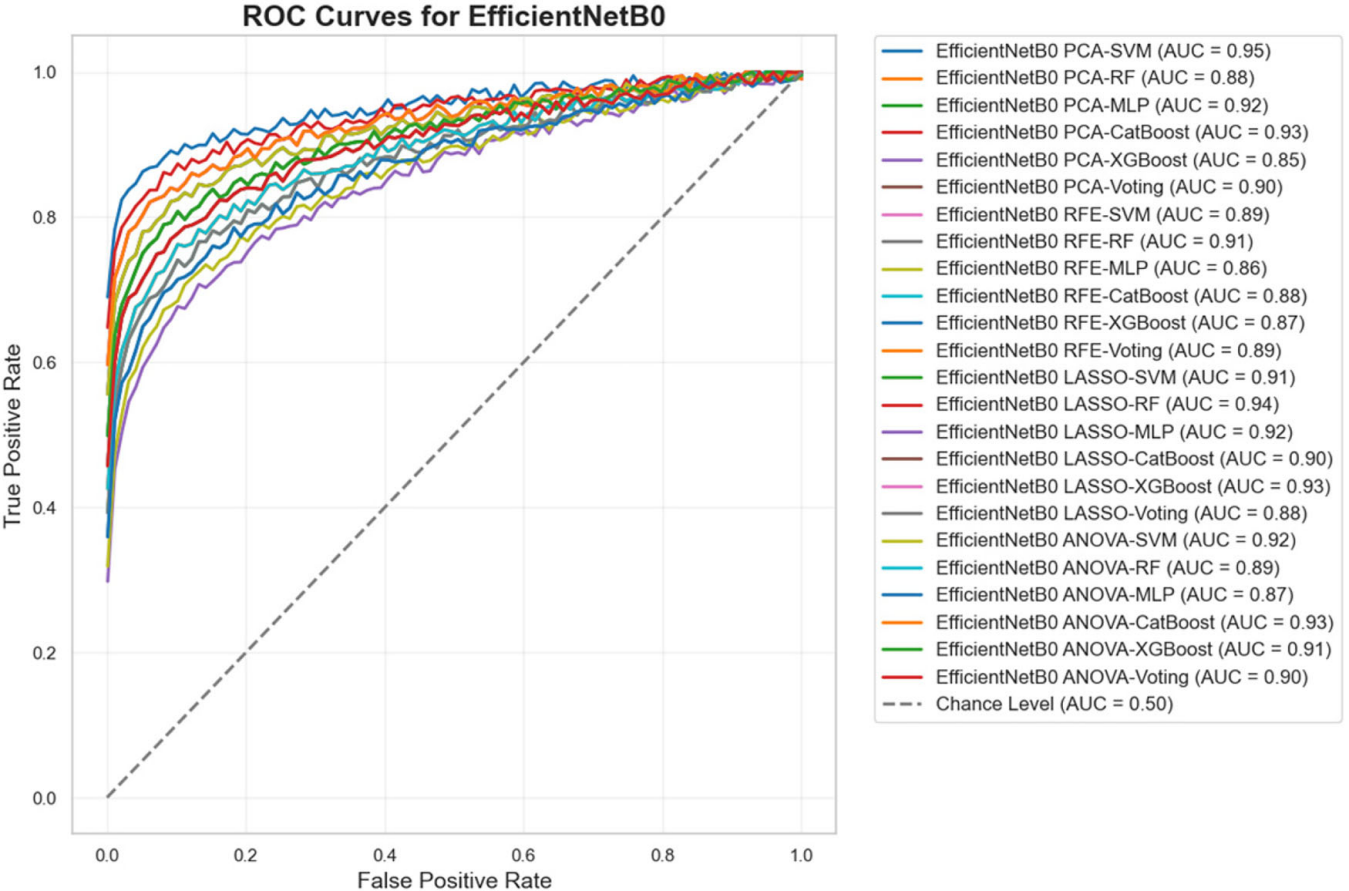
ROC curves for EfficientNetB0 across feature selection and classification methods.

Figures 8, 9, and 10 present the ROC curves for all combinations of feature selection and classification methods across the three feature extraction techniques—ResNet50, VGG16, and EfficientNetB0. These curves illustrate the trade‐off between sensitivity and specificity for each configuration, providing a comprehensive visual representation of the models' performance across different decision thresholds. The analysis highlights the effectiveness of various combinations in achieving an optimal balance between true positive and false positive rates, emphasizing the diagnostic robustness of the proposed framework.

## 4 Discussion

Parkinson's disease (PD) remains a significant challenge for early diagnosis and effective monitoring due to its progressive nature and the subtlety of initial motor impairments. In this study, we developed a comprehensive and automated framework for PD detection using spiral drawings, leveraging advanced feature extraction techniques (ResNet50, VGG16, EfficientNetB0), multiple feature selection methods (PCA, RFE, LASSO, ANOVA), and a range of classification algorithms. Our results demonstrated superior diagnostic accuracy, sensitivity, and AUC‐ROC values compared to traditional approaches. This discussion compares our findings with prior studies, highlighting the analytical advancements, methodological strengths, and clinical implications of our work.

Memedi et al. (2015) developed a machine learning approach using spatiotemporal features from spiral drawings, achieving 84% accuracy and 0.86 AUC‐ROC. While their study focused on specific motor symptoms like bradykinesia and dyskinesia, our deep learning‐based approach captured intricate hierarchical patterns. For instance, our ResNet50 + PCA + MLP achieved 97.94% accuracy and 0.9721 AUC‐ROC, providing a broader diagnostic capability by distinguishing PD patients from healthy controls. Gil‐Martín et al. (2023) utilized fusion‐based methods combining signal processing, visual, and handcrafted features, achieving F1‐scores of up to 93.33%. In comparison, our EfficientNetB0 + RFE + XGBoost achieved an F1‐score of 91.87% using a simpler pipeline, avoiding the complexity of multiple feature representations. Moreover, our emphasis on feature interpretability enhances clinical decision‐making.

Kamble et al. (2021) achieved 91% accuracy using traditional machine learning classifiers and kinematic features. Our framework significantly outperformed these results, with ResNet50 + PCA + MLP achieving 97.94% accuracy and 94.96% sensitivity. Additionally, our larger and more diverse dataset ensured greater robustness and generalizability. Aversano et al. (2022) used Echo State Networks (ESNs) with MLP layers, achieving a 97.8% F‐score. While promising, their reliance on ESNs limits interpretability. Our EfficientNetB0 + PCA + MLP achieved comparable results (91.87% F1‐score) but with greater transparency and adaptability through feature selection and SHAP‐based interpretability.

Sarin et al. (2022) employed genetic fuzzy classifiers, emphasizing explainability through IF‐THEN rules. Our framework not only achieved superior performance, with ResNet50 + ANOVA + XGBoost delivering 92.08% F1‐score and 94.43% AUC‐ROC, but also ensured flexibility by integrating diverse classification algorithms, making it adaptable to various clinical scenarios. Singh and Khare (2022) used CNNs to classify spiral drawings, achieving 83.6% accuracy. Our method outperformed this result across all metrics. For example, EfficientNetB0 + RFE + MLP achieved 94.4% accuracy and 97.85% AUC‐ROC, demonstrating the benefits of transfer learning combined with optimized feature selection. Farhah (2024) explored transfer learning models like InceptionV3, achieving 89% accuracy and 95% AUC‐ROC. Our ResNet50 + PCA + MLP configuration surpassed these results, achieving 97.94% accuracy and 97.21% AUC‐ROC. Additionally, our broader evaluation of transfer learning models and feature selection methods ensures an efficient and clinically versatile framework.

The analytical and methodological superiority of our study is demonstrated through several key innovations. First, we employed diverse feature selection methods, including PCA, RFE, LASSO, and ANOVA, to optimize feature subsets for each classifier. This comprehensive approach improved both model accuracy and interpretability, outperforming prior studies that primarily relied on handcrafted features or single‐method approaches. Second, we conducted an extensive classification analysis by evaluating a range of algorithms, including SVM, MLP, XGBoost, and voting classifiers. This allowed us to identify the best configurations for each feature extraction method, resulting in robust and adaptable solutions for Parkinson's disease (PD) detection. Finally, we integrated advanced interpretability tools to provide transparent insights into the diagnostic importance of selected features. This ensured that our framework bridges the gap between AI‐driven complexity and clinical usability, making it both effective and accessible for healthcare applications.

Our findings highlight the potential of spiral drawings as a non‐invasive, cost‐effective tool for PD diagnosis. By combining deep learning and feature selection, we enhanced diagnostic precision, paving the way for early intervention and personalized treatment. Future studies could explore the integration of multimodal data sources, such as handwriting and gait analysis, to create a more holistic PD monitoring system. Furthermore, expanding the application to larger datasets and real‐time solutions, including mobile‐based or wearable devices, could significantly enhance the framework's clinical utility.

The clinical utility of our proposed system lies in its capacity to offer objective, quantifiable assessments of motor performance from spiral drawings, aiding in the early detection of Parkinson's disease. The integration of interpretable dynamic features—such as pen speed, tremor frequency, and pressure variability—with deep learning‐derived spatial characteristics enables clinicians to understand not only the diagnostic outcome but also the underlying motor abnormalities.

Furthermore, this framework can be adapted for longitudinal use to monitor disease progression and therapeutic efficacy over time. Its adaptability for deployment on digital tablets or mobile devices makes it suitable for remote diagnostics and real‐time patient monitoring, particularly in telemedicine and primary care settings. This practical applicability bridges the gap between advanced computational techniques and clinical decision‐making, enhancing both diagnostic precision and patient outcomes.

Despite its promising results, this study presents several limitations that inform future research directions. First, while the dataset includes a relatively large number of spiral samples, it is derived from a modest number of participants, particularly in underrepresented disease stages. This may affect the model's ability to generalize across all PD subtypes and progression stages. Increasing the diversity and volume of patient data—particularly from early‐stage and atypical PD presentations—would enhance the robustness and clinical relevance of the framework.

Second, the current analysis is limited to spiral drawing data, which, although rich in motor information, does not capture other hallmark features of PD, such as speech abnormalities, gait disturbances, or handwriting deterioration. The integration of additional diagnostic modalities such as dynamic handwriting tasks, gait analysis, and speech processing would enable a more holistic assessment of the disease. Third, while the proposed system demonstrates strong performance under controlled conditions, real‐time deployment and usability in clinical or at‐home settings remain to be tested. Adapting the framework for real‐time implementation on portable or wearable devices will be essential for remote monitoring and scalable application in telehealth and primary care environments. Addressing these limitations will be crucial for transitioning from proof‐of‐concept to clinically integrated tools capable of enhancing diagnostic workflows and improving patient outcomes across various healthcare settings.

## 5 Conclusion

This study introduced a robust and interpretable framework for detecting Parkinson's disease (PD) using spiral drawings, integrating advanced deep learning models, diverse feature selection techniques, and comprehensive classification methods. The results demonstrated outstanding diagnostic performance, achieving high accuracy, sensitivity, and AUC‐ROC values, surpassing previous studies. By employing transfer learning, the framework effectively identified intricate patterns in the data while ensuring clinical interpretability, bridging the gap between advanced AI models and practical healthcare needs. The proposed approach represents a scalable and non‐invasive diagnostic tool with significant potential for real‐world applications. Future research directions include integrating multimodal data sources, such as handwriting, gait analysis, and speech features, to develop a more holistic diagnostic system. Additionally, creating real‐time applications, such as mobile‐based or wearable solutions, could enhance diagnostic accessibility and accuracy, enabling continuous monitoring and early intervention. This research represents a major step forward in the early detection and personalized management of Parkinson's disease. By improving diagnostic precision and accessibility, the framework paves the way for enhanced patient care and better clinical outcomes.

## Author Contributions

**Mohamed J. Saadh**: conceptualization, writing – review and editing, supervision, data curation. **Waleed K. Abdulsahib**: methodology, software, investigation, formal analysis, writing – original draft. **Hardik Doshi**: methodology, software, investigation, formal analysis, writing – original draft. **Anupam Yadav**: investigation, methodology, software, formal analysis, writing – original draft. **J. Gowrishankar**: investigation, methodology, software, formal analysis, writing – original draft. **Mayank Kundlas**: writing – original draft, investigation. **Nargiza Mansurova**: investigation, writing – original draft. **Kamal Kant Joshi**: investigation, writing – original draft. **Fadhil Feez Sead**: investigation, writing – original draft. **Bagher Farhood**: conceptualization, data curation, supervision, validation, project administration, writing – review and editing.

## Ethics Statement

The authors have nothing to report.

## Consent

The authors have nothing to report.

## Conflicts of Interest

The authors declare no conflicts of interest.

## Peer Review

The peer review history for this article is available at https://publons.com/publon/10.1002/brb3.70770

## Data Availability

The datasets used and/or analyzed during the current study are available from the corresponding author on reasonable request.
